# Novel insights on effect of atrioventricular programming of biventricular pacemaker in heart failure – a case series

**DOI:** 10.1186/1476-7120-4-38

**Published:** 2006-10-16

**Authors:** Tasneem Z Naqvi, Asim M Rafique

**Affiliations:** 1 Cardiac Non-Invasive Laboratory, Division of Cardiology, Department of Medicine, Cedars-Sinai Medical Center, Los Angeles, California, USA

## Abstract

**Background:**

Echocardiography plays an integral role in the diagnosis of congestive heart failure including measurement of left heart pressure as well as mechanical dyssynchrony.

**Methods:**

In this report we describe novel therapeutic uses of echo pulsed wave Doppler in atrioventricular pacemaker optimization in patients who had either not derived significant symptomatic benefit post biventricular pacemaker implantation or deteriorated after deriving initial benefit. In these patients atrioventricular optimization showed novel findings and improved cardiac output and symptoms.

**Results:**

In 3 patients with Cheyne Stokes pattern of respiration echo Doppler showed worsening of mitral regurgitation during hyperpneac phase in one patient, marked E and A fusion in another patient and exaggerated ventricular interdependence in a third patient thus highlighting mechanisms of adverse effects of Cheyne Stokes respiration in patients with heart failure. All 3 patients required a very short atrioventricular delay programming for best cardiac output. In one patient with recurrent congestive heart failure post cardiac resynchronization, mitral inflow pulse wave Doppler showed no A wave until a sensed atrioventricular delay of 190 ms was reached and showed progressive improvement in mitral inflow pattern until an atrioventricular delay of 290 ms. In 2 patients atrioventricular delay as short as 50 ms was required to allow E and A separation and prevent diastolic mitral regurgitation. All patients developed marked improvement in congestive heart failure symptoms post echo-guided biv pacemaker optimization.

**Conclusion:**

These findings highlight the value of echo-guided pacemaker optimization in symptomatic patients post cardiac resynchronization treatment.

## Background

Echocardiography has become the gold standard for non invasive assessment of diastolic function [1]. Besides detecting left ventricular (LV) relaxation abnormality, pulsed wave (PW) Doppler echocardiography allows assessment of LV end diastolic and left atrial pressures accurately [2,3]. More recently tissue Doppler imaging (TDI) has been shown to allow detection of mechanical asynchrony in patients with congestive heart failure (CHF) and predict improvement in response to cardiac resynchronization treatment (CRT) [4-8]. Patients with advanced CHF have concomitant diastolic dysfunction to varying degree and are very dependent on atrial output to prevent pulmonary venous congestion and maintenance of effective cardiac output. Atrial flutter, tachycardia induced E and A fusion and diastolic mitral regurgitation (MR) markedly compromise ventricular diastolic filling thereby leading to increase in left atrial pressure. Biventricular (biv) pacing has become an effective method to improve diastolic filling as well as LV ejection times [9]. In-coordinated diastolic filling may be one the important reasons why a significant number of patients do not improve or even deteriorate after CRT despite improvement in ventricular synchrony [8]. Since AV delay affects atrial contribution to LV filling, manipulation of AV delays may further improve diastolic filling post biv pacemaker implantation in 12–25% of subjects [9,10]. PW Doppler is able to detect acute changes in response to pacemaker programming allowing this simple non invasive technique to be used during biv pacemaker programming. Indeed tailored echocardiography guided AV programming has been shown to cause incremental improvement in cardiac function and functional class in patients who undergo CRT [1,11-13]. In this report we describe our experience with AV optimization by case examples of patients where optimal AV delay varied considerably from the standard AVD of 120 ms that is often programmed empirically post CRT. These case examples are derived from 200 consecutive AV optimizations we performed at our center between Jan 2004 to March 2006. Several patients with findings similar to those presented are not discussed. Besides highlighting the role of echo Doppler in AV optimization, these case examples gave insight into the mechanism of CHF symptoms post CRT. Echo Doppler evaluation of mitral inflow, left ventricular (LV) outflow and pulmonary vein inflow during pacemaker programming was performed using Vivid 7, GE Vingmed ultrasound system using conventional methods. Data was averaged from 5 cardiac cycles for each AV delay.

### Effect of Cheyne Stokes respiration on diastolic filling

**Figure 1:**
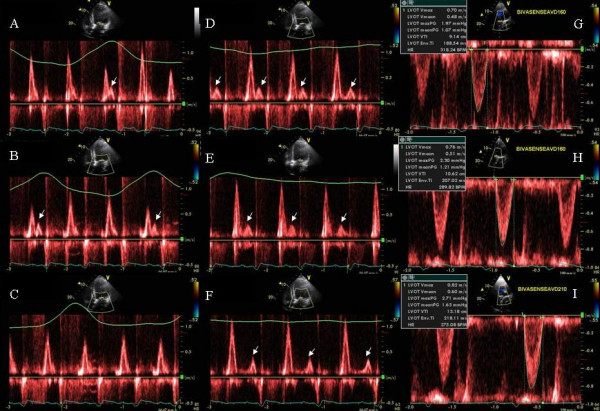
Figure shows respirogram (green line at the top of each panel), electrocardiogram (green signal at the bottom of each panel) and mitral inflow pulsed wave Doppler during hyperpneac phase (panels A, B and C), progressing gradually to a hypopneac (D) and then an apneac phase (panels E and F). Progressive decrease in heart rate, from 94 bpm in panel A to 65 bpm in panel F, is shown as hyperpneac phase slows into an apneac phase. This is associated with progressive separation of mitral inflow E and A waves. Regular A waves are evident in panels D, E and F, whereas A waves with minimal E and A separation occur when diastolic mitral inflow coincides with peak inspiration during the hyperneac phase as shown in panels A and B. White arrows depict mitral inflow A waves. Panels G, H and I show the LV outflow tract pulsed wave Doppler velocity and VTI which improves progressively from 9.14 cm during early hyperpneac phase at heart rate of 93 bpm to 10.62 cm during mid respiratory phase at a heart rate of 84 bpm and finally to 13.18 cm at heart rate of 66 bpm during the apneac phase.

**Figure 2:**
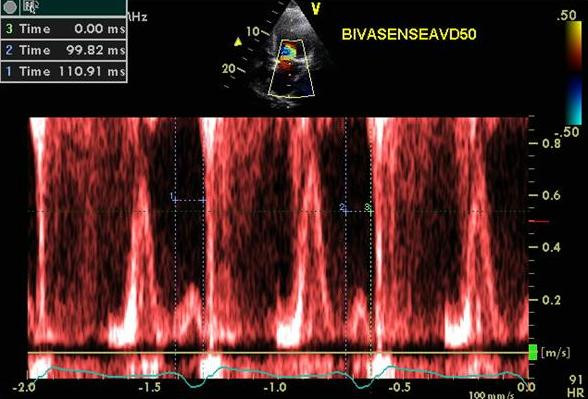
Figure shows the effect of shortening of AVD from 160 ms to 50 ms. E and A fusion seen during the hyperpneac phase of respiration at a heart rate of ≥ 90 bpm (Figure 1, panels A and B) when the AVD was programmed at 160 ms is no longer seen.

A 51 year old African American obese male with a history of non-ischemic dilated cardiomyopathy improved CHF symptoms from NYHA class III to class II after biv pacemaker implantation. Patient developed recurrent CHF with NYHA class III symptoms and an LVEF of 20% 8 months post CRT. On interrogation there was normal sinus rhythm and pacemaker was programmed in the DDD mode, AV delay of 160 ms and a VV delay of 0 ms. Mitral inflow PW Doppler and respirogram revealed cyclic variation of heart rate from 56 to 94 beats per minute during Cheyne Stokes respiratory cycle. Post apneac hyperpnea was associated with tachycardia (Figure 1A–C) and hypopnea and apnea were associated with a progressively decreasing heart rate (Figure 1D–F). PW Doppler of mitral inflow showed E and A fusion during hyperpneac and tachycardic phase (Figure 1A–C) and good E and A separation during the bradycardia associated with hypopnea and apnea (Figure 1D–F). LV velocity times integral (VTI) improved from 9 cm to 13 cm between the hyperpneac to the apneac phases (Figure 1G–I). Shortening the AV delay to 50 ms resulted in E and A separation throughout the respiratory cycle and abolished E and A fusion during hyperpnea (Figure 2). BNP improved from 1241 to 950 pg/ml post CRT and to 877 pg/ml 3 days post AV optimization. Pt was advised continuous positive airway pressure (CPAP) during sleep.

**Figure 3:**
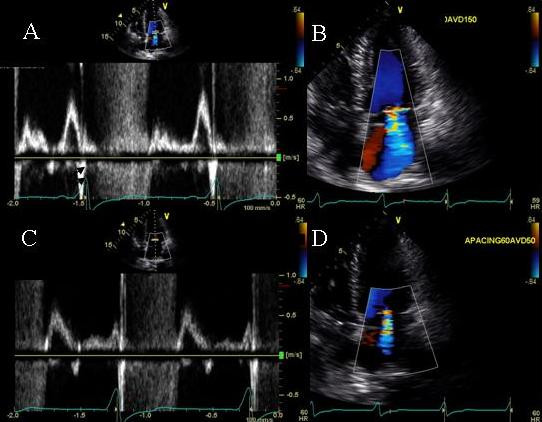
Figure shows the effect of AVD on mitral regurgitation severity. Mitral inflow pulsed Doppler (A) and color Doppler showing mitral regurgitation in the apical 4 chamber view (B) at a paced AV delay of 150 ms and mitral inflow pulsed Doppler (C) and color Doppler showing mitral regurgitation severity in the apical 4 chamber view (D) at paced AV delay of 50 ms. Diastolic mitral regurgitation is seen at an AV delay of 150 ms (black arrowheads, A). Note significant mitral regurgitation in panel B which decreased in panel D at short AV delay.

### Effect of Cheyne Stokes respiration on mitral regurgitation

**Figure 4:**
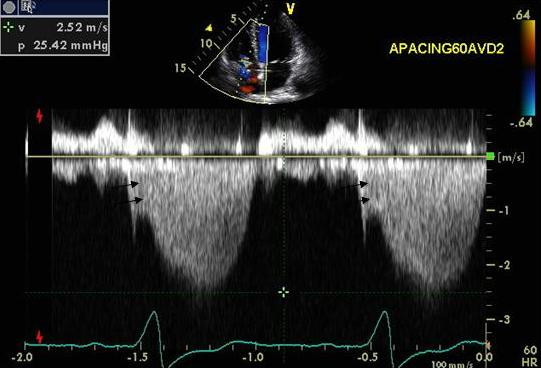
Continuous wave Doppler across tricuspid valve obtained during paced atrial rate of 60 bpm and atrioventricular delay of 220 ms showing significant diastolic tricuspid regurgitation (white arrow head).

**Figure 5:**
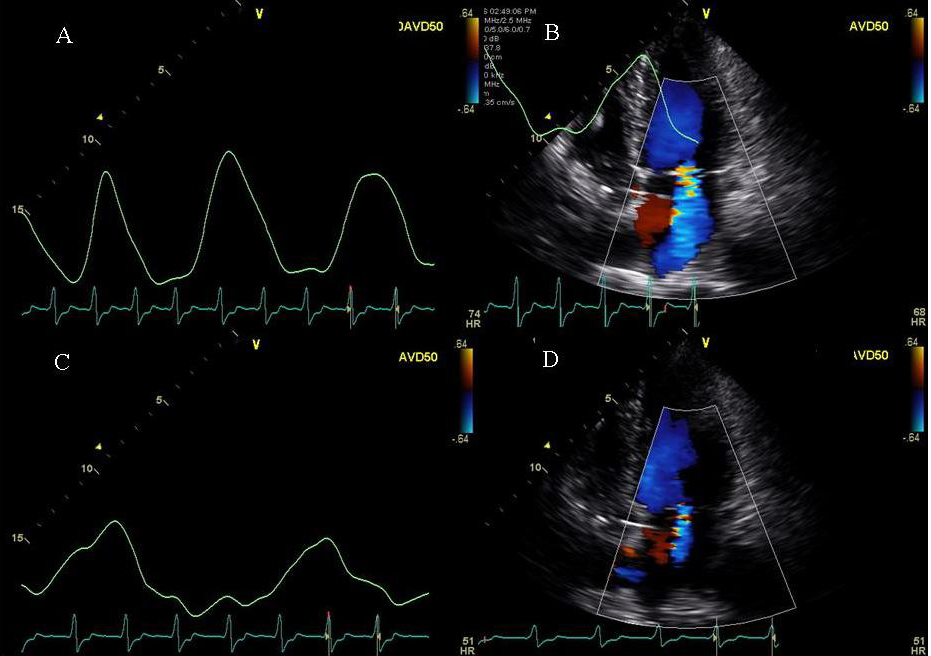
Figure shows the effect of respiratory phase of Cheyne Stokes breathing on mitral regurgitation severity in a 76 year old female with biv pacemaker. Tachypnea and hyperpnea (green respirogram, A) is associated with increased heart rate between 68–74 bpm (A and B) and significant mitral regurgitation (B), whereas bradypnea (green respirogram, C) is associated with bradycardia (51 bpm, C and D) and a marked reduction in mitral regurgitation severity (D). All recordings were performed during sensed AVD of 50 ms and back up atrial paced rate of 40 bpm.

A 76 year old Caucasian female developed increasing shortness of breath and NYHA class III symptoms for 8 weeks. Patient had a history of RV pacemaker implantation for a complete heart block followed by development of CHF class II-III. RV pacemaker was upgraded to a biv pacemaker that led to improvement in symptoms for about 7 months. A-pacing at 60 bpm and paced and sensed AVD of 170 and 150 ms respectively and LV pre-excitation of 20 ms were programmed. Moderate to severe mitral regurgitation (MR) with onset in diastole was seen at these settings (Figure 3A and 3B). Significant diastolic tricuspid regurgitation was also seen that got accentuated at AVD of 220 ms (Figure 4). AVD of 50 ms lead to marked reduction in MR severity (Figure 3C and 3D). Subsequently as the patient drifted off to sleep during the study, Cheyne Stokes pattern of respiration was seen with an increase in MR severity at this AVD of 50 ms during hyperpnea and increased heart rate (Figure 5A and 5B) and reduction in MR severity during hypopnea and bradycardia (Figure 5C and 5D). VV optimization required LV pre-excitation of 10 ms. To prevent right ventricular diastolic tricuspid regurgitation, AVD was lowered further to 40 ms with excellent results. At the end of the study patient felt an immediate improvement and performed a brisk 3 minute hall walk with a marked improvement in symptoms. Pt was advised CPAP during sleep.

### Effect of Cheyne Stokes respiration on ventricular interdependence

**Figure 6:**
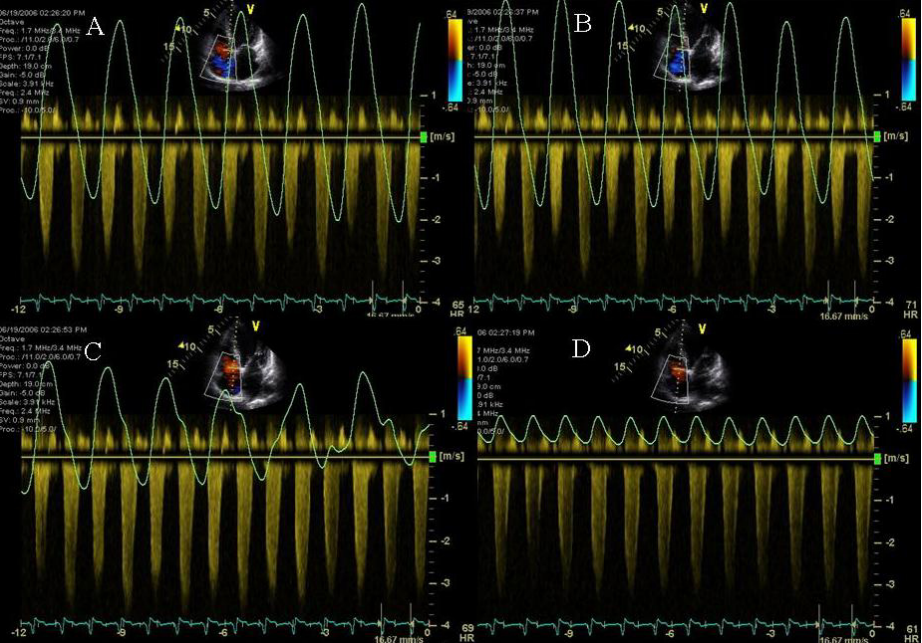
CW Doppler across tricuspid inflow is shown during hyperpneac phase (A and B) and during hypopnea (C and D). Green graph in each panel is the respirogram. Note tricuspid regurgitation velocity increase during expiration and decrease during inspiration in A and B that is decreased at the onset of hypopnea in C and abolished during hypopnea in D.

**Figure 7:**
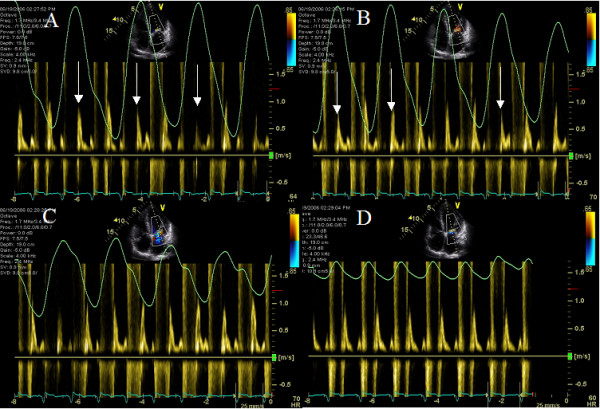
PW Doppler across mitral inflow is shown during hyperpneac phase (A and B) and during hypopnea (C and D). Green graph in each panel is the respirogram. Note mitral inflow velocity decrease during each inspiration (white arrows, A and B) and increase during each expiration. This exaggerated respiratory change is decreased at the onset of hypopnea in C and abolished during hypopnea in D.

A 72 year old African American female with ischemic cardiomyopthy and prior left cerebrovascular accident and right hemiparesis presented with CHF and NYHA class III symptoms 1 year after biV pacing. LVEF was 25%, there was mild MR and peak pulmonary artery systolic pressure was 50 mm Hg. Cheyne Stokes respiratory pattern was seen on respirogram. Hyperpnea was associated with decreased RV-RA gradient during each inspiration (Figure 6A and 6B). In addition decrease in mitral inflow E and A wave velocities was seen during each inspiration during the hyperpneac phase (Figure 7A and 7B). These changes in tricuspid CW Doppler (Figure 6C and 6D) and mitral inflow PW Doppler (Figure 7C and 7D) were abolished during hypopneac phase of respiration. Heart rate of upto 70 bpm was seen following hyperpnea and upto 60 bpm following hypopnea (Figures 6 and 7). Complete biv capture was seen at an AVD of 120 ms and below and premature mitral valve closure at an AVD of 50 ms. Optimal AVD was determined to be 100 ms and optimal VV delay to be LV pre-excitation of 20 ms. Pt was advised CPAP during sleep.

### Effect of a long atrioventricular delay on late diastolic filling

**Figure 8:**
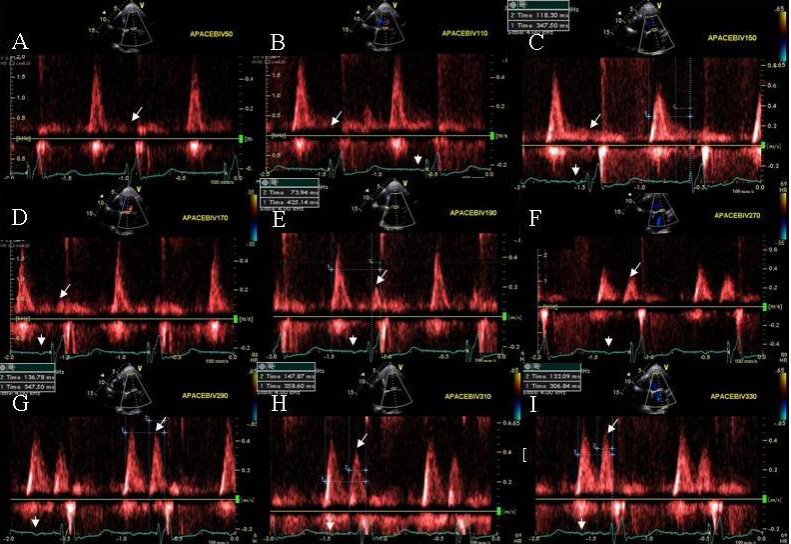
Pulsed wave mitral inflow Doppler tracings obtained at AVD of 50 (A), 110 (B), 150(C), 170(D) 190(E), 270(F), 290(G), 310(H), and 330ms (I). Note absent mitral inflow A waves (white arrows) in panels A through D until an AV delay of 190 ms (E) is reached. Significant increase in atrial velocity and duration is seen at an AVD of 290 ms (Panel G). Further increase in AVD caused E and A approximation (H and I). White arrow heads next to EKG signals in each panel point at the p waves on electrocardiogram displayed on ultrasound system during progressively increasing AVD.

**Figure 9:**
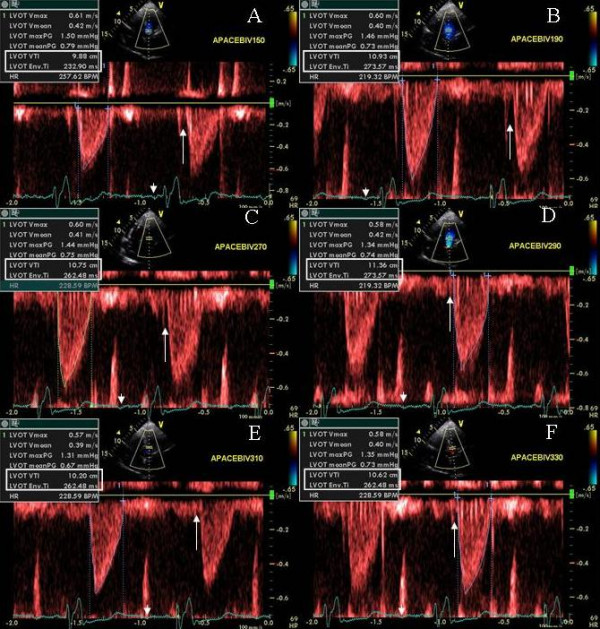
Effect of AVD change on stroke volume. Note progressive shortening of isovolumic contraction time (white arrows) and progressive increase in LV outflow tract VTI and ejection time until an AVD of 290 ms (A-D) is reached. Further increase in AVD leads to increase in isovolumetric contraction time and decrease in VTI (E and F). White rectangles at top left of each panel highlight data obtained from measurement of each individual beat.

A 73 Caucasian male was referred for bi-V pacemaker optimization 7 months post CRT for ischemic cardiomyopathy. Symptoms of CHF improved initially, however worsening shortness of breath and fatigue occurred 5 months later to NYHA class III symptoms. BNP was 1040 pg/ml. The device was programmed in the DDD mode, with sensed AV delay of 150 ms and paced AV delay of 160 ms. PW Doppler showed absent mitral inflow A waves at baseline pacemaker settings despite presence of sinus rhythm (Figure 8A–D). AV delay was progressively increased from 50 ms to 330 ms in 10 ms increments with continued bi-V capture. No mitral inflow A wave seen until an AV delay of 190 ms (Figure 8, white arrows, 8E) and A wave velocity and duration showed a progressive increase upto an AV delay of 290 ms (Figure 8, white arrows 8F and 8G). This was accompanied by a progressive improvement in LV velocity times integral (VTI) at AVD of 190 ms until AVD of 290 ms (Figure 9B, C and 9D). There was a concomitant reduction in isovolumic contraction time between AVD of 150 ms to 290 ms (Figure 9B, C and 9D). Further increase in AV delay to 310 ms and 330 ms led to a decrease in LV VTI and an increase in isovolumic contraction time (Figure 9E and 9F). Next VV optimization was performed. RV-LV offset of 5 ms was selected. At the end of pacemaker programming the patient felt immediate improvement and performed a brisk 6 minute hall walk without shortness of breath. At follow up assessment at 4 weeks, the patient was is NYHA class II and BNP level had reduced to 600 pg/ml.

### Effect of a very short atrioventricular delay on diastolic mitral regurgitation

**Figure 10:**
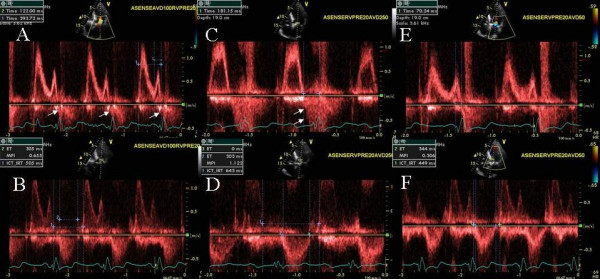
Top panels show the pulsed wave mitral inflow Doppler at AVD of 100, 250 and 50 ms and bottom panels show mitral inflow and aortic outflow PW Doppler at the same settings. Marked E and A fusion and diastolic mitral regurgitation (white arrows) is shown in panel B along with reduced aortic ejection time and marked increase in myocardial performance index as shown in panel D. A marked improvement in mitral diastolic filling time and E and A separation is shown in panels A and E at an AVD of 100 and 50 ms respectively and a corresponding improvement in LV ejection time as well as myocardial performance index as shown in panels B and F. Note that diastolic mitral regurgitation is still present at an AVD as short as 100ms (white arrows panel A).

An 83-year-old Caucasian male with a history of ischemic cardiomyopathy, CHF and NYHA class II symptoms underwent biv pacemaker implantation. Sensed AVD was 100 ms and RV-LV offset was 20 ms. Routine pre-discharge optimization revealed late diastolic MR (Figure 10A-white arrows) and myocardial performance index of 0.66 (Figure 10B) at an AVD of 100 ms. Increase in AVD to 250 ms caused marked E and A fusion, worsening of diastolic MR (Figure 10C) and increase in myocardial performance index to 1.12 (Figure 10D). Shortening the AVD to 50 ms increased mitral inflow filling time, abolished diastolic MR (Figure 10E) and improved myocardial performance index to 0.31 (Figure 10F). At 1 month there was an improvement in NYHA class from class II to class I and in LVEF improved from 35% to 43%.

### Effect of a very short atrioventricular delay on E and A separation

**Figure 11:**
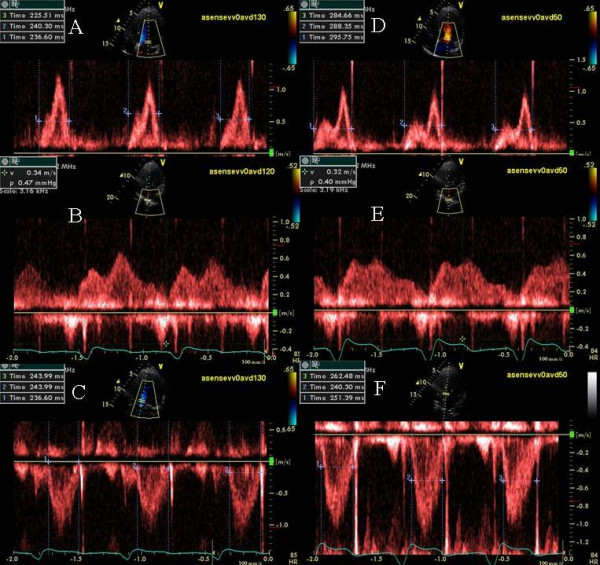
Effect of shortening of AVD on diastolic filling. A, B and C are mitral inflow, pulmonary vein inflow and aortic outflow PW Doppler tracings respectively obtained at an AVD of 130 ms and D, E and F are corresponding PW Doppler tracings obtained at an AVD of 50 ms. Note marked improvement in mitral inflow filling time (from 234 ms to 289 ms), change in pulmonary vein flow from diastolic dominant to systolic dominant along with decrease in atrial reversal velocity (from 34 to 32 cm/s) and improved LV ejection duration (from 242 to 251 ms).

A 67 year old Caucasian female underwent biv pacemaker implantation for non ischemic dilated cardiomyopathy, severe mechanical dyssynchrony, left bundle branch block, NYHA class III and LVEF of 35%. She was referred for persistent shortness of breath upon exertion post CRT. Programmed AVD of 130 ms showed marked E and A fusion (Figure 11A) and diastolic dominant pulmonary vein flow (Figure 11B). Shortening the AV delay to 50 ms led to marked improvement in mitral inflow PW Doppler pattern (Figure 11F), change in pulmonary vein flow to systolic dominant pattern (Figure 11E) and improvement in LV ejection period (Figure 11C and 11D). Patient developed marked improvement in symptoms immediately post optimization.

## Discussion

Our findings describe the utility of AV pacemaker optimization using PW echocardiography in optimizing diastolic mitral inflow in patients who remain symptomatic post CRT. Our report also highlights the mechanisms of adverse effects of Cheyne Stokes pattern of respiration on cardiac performance.

Other investigators have reported the incremental improvement in cardiac function and functional class by echocardiographic guided pacemaker optimization in patients who undergo CRT [1-3]. Studies on biv pacemaker optimization using AVD are summarized in Table 1. These studies attempted AV optimization using AVD of 40–250 ms and described effective AV delay ranging from 70 ms to 250 ms. In this report, we describe patients requiring AVD ranging from 40–290 ms. Our series illustrates that optimization of AVD under echocardiographic guidance may lead to a significant improvement in cardiac output and should be considered in patients who have not derived benefit from CRT or who have deteriorated after deriving initial benefit from CRT.

**Table 1:** Published Studies on Biventricular Pacemaker Programming

**Study**	**N**	**Short AVD**	**Long AVD**	**Optimal AVD**	**Criteria**
Shawnee NS [21]	20+20	60	200		Aortic VTI
Inoue N [22]	5			70–100	Mitral PW Doppler
Braunschweig F [23]	1	110	250	190	Intracardiac Hemodynamic Monitor
Santos JF [24]	7	80	QRS Fusion	120–170 vs. 110–190	PW Mitral vs Impedance Cardiography
Meluzin J [25]	18			120–180	Rt. Heart Cath + Mitral PW Doppler
Butter C [26]	57	40	190		Invasive Aortic Pressure + FPPG
Braun MU [27]	24	60	200	80–180	Impedance Cardiography S+ VTI
Ishikawa T [28]	1	50	110	70–100	Mitral PW Doppler + Aortic VTI
Scharf C [29]	36	40	200		Aortic VTI

AVD = atrioventricular delay, CO = cardiac output. VTI = velocity times integral, FPPG = finger pulse plethysmography.

Shortening of AV delay has been shown to reduce diastolic MR in patients with right sided pacemaker [14], and causes hemodynamic improvement in patients with advanced LV systolic dysfunction by optimizing mitral inflow filling [15]. We describe the effect of shortening of AVD in reducing MR in symptomatic patients with biv pacemaker. In addition we report the beneficial effects of a short AVD in systematic patients with biv pacemaker who have Cheyne Stokes respiration.

Central sleep apnea is common among patients with CHF, being present in 30–40% of patients [16]. It contributes to increased mortality among patients with CHF [17] due to changes in sympathetic activity with respiratory phases [18] as well as by secondary pulmonary hypertension and right ventricular dysfunction. Rhythmic oscillations in AV node refractoriness occurs with Cheyne Stokes respiration such that a short AV node refractoriness occurs during hyperpnea with a lesser degree of concealed conduction [19,20]. This explains findings in all 3 of our patients with Cheyne Stokes respiration in whom tachycardia occurred during hyperpneac phase and bradycardia during bradypneac and apneac phases of respiration. Increased heart rate and blood pressure on the average show about a 10-second delay because sympathetic system modulates heart rate at lower frequencies than parasympathetic responses which are greatly diminished in patients with heart failure. Thus our patients showed a systematic delay between onset of respiratory phases and change in heart rate. One of our patients with this respiratory pattern also showed mitral inflow E and A fusion causing impairment in diastolic filling during hyperpneac phase of Cheyne Stokes respiration, another showed an increase in MR severity and another showed exaggerated mitral and tricuspid inflow respiratory variation during hyperpneac phase of respiration. These observations highlight the additional mechanisms whereby Cheyne Stokes respiration causes adverse cardiac effects including worsening of heart failure symptoms as well as increased mortality. Our observations suggest that presence of Cheyne Stokes respiration should be evaluated in patients who are referred for pacemaker optimization due to persistent CHF post biv pacemaker implantation.

## Limitations

Ours is not a consecutive series of patients, rather a collection of patients in whom echo Doppler during AV optimization allowed novel observations that provide insight into the mechanism of failure of CRT and adverse effects of Cheyne Stokes respiration. A systemic study is needed to prospectively evaluate prevalence of Cheyne Stokes respiration in symptomatic patients post CRT and effect of AVD optimization in this group.

## Conclusion

We report 3 separate mechanisms whereby Cheyne Stokes respiration can contribute to worsening of heart failure symptoms in patients with CHF post CRT. These include compromised diastolic filling during hyperpneac phase of respiration, increase in diastolic mitral regurgitation during hyperpneac phase and development of a constrictive physiology with enhanced ventricular interdependence. A short AVD helped improve diastolic filling, reduce diastolic MR and reduce ventricular interdependence in all 3 patients. We also report the use of short AVD in abolishing diastolic MR in patients without Cheyne Stokes respiration as well as improvement in diastolic filling by an extraordinarily long AVD in a patient with heart failure post CRT. Our findings extend the use of echo Doppler in evaluation of patients who remain symptomatic post CRT as well as in determining optimal AVD.

## Abbreviations

AVD = Atrioventricular Delay

CHF = Congestive Heart Failure

CRT = Cardiac Resynchronization Treatment

CPAP = Continuous Positive Airway Pressure

LVEF = Left Ventricular Ejection Fraction

LV = Left Ventricle (Ventricular)

MR = Mitral Regurgitation

NYHA = New York Heart Association

PW = Pulsed wave

RA = Right Atrium

RV = Right Ventricle (Ventricular)

VTI = Velocity Times Integral

## Competing interests

The author(s) declare that they have no competing interests.

## Authors' contributions

TZN conceptualized the design of the study, imaging methodology and interpreted data during acquisition. AMR analyzed echocardiographic data and helped to draft the manuscript. All authors read and approved the final manuscript.
